# Reviewed article: Pimenta IT, Marsico EFC, Souto APM, Geraldo MCHM, Amaral LF, Duffrayer KM, Ribeiro CLP, Aguilar GMO. Vigilância em saúde em eventos de massa: relato da experiência do Centro de Informações Estratégicas em Vigilância em Saúde da cidade do Rio de Janeiro, 2024. Epidemiol Serv Saude. 2025:34;e20250212

**DOI:** 10.1590/S2237-96222025v34e20250212.c

**Published:** 2025-09-29

**Authors:** Marilia Mastrocolla de Almeida Cardoso

**Affiliations:** 1 Ministério da Saúde, Brasília, DF, Brazil

## First round comments

Prezados autores, seguem algumas sugestões complementares ao que foi pontuado pelos revisores, no sentido de fortalecer o conteúdo do manuscrito e o objetivo de contribuir para o aprimoramento contínuo das práticas de vigilância em eventos de massa, fortalecendo a capacidade de resposta em âmbito nacional:

1) Na introdução : será importante explicar e mencionar a importância das ações de vigilância. Porque elas existem, quais são os princípios e métodos norteadores, o que são serviços de vigilância, porque eles são acionados nestes contextos, qual política nacional que descreve essas ações entre outras informações. Essas informações são importantes uma vez que a RESS possui um público leitor diverso.

2) No método:

- Fornecer mais detalhes sobre o serviço e a equipe.

- Como a estratégias de detecção digital da vigilância baseada em eventos está citada pela primeira vez no método, é importante trazer referência sobre ela e explicar brevemente como ela é aplicada e do que ela é composta.

- Explicar com mais detalhes a realização dos grupos focais (quem conduz, quem participa, o que se discute, como é realizado e quais informações são utilizadas a partir dos resultados) 

- Como o treinamento da equipe é realizado?

3) Nos resultados

- Quantos postos de saúde foram visitados?

- Como foram realizadas as avaliações: “Também foram avaliadas as ações implementadas no pré, durante e pós-evento para aprimorar as estratégias de detecção e resposta do sistema de vigilância, o que mobilizou a Secretaria Municipal de Saúde a incluir, nas discussões e planejamento das ações para os próximos eventos de massa, iniciativas voltadas à prevenção e monitoramento dos impactos das condições climáticas na saúde dos participantes, como altas temperaturas e tempestades, além da gestão de riscos relacionados a desastres.”

4) Na discussão:

- Poderiam explorar mais “O planejamento e a resposta à disseminação de doenças transmissíveis e ocorrência de eventos e emergências de saúde pública, decorrentes do intenso fluxo nacional e internacional de pessoas também foram discutidos em outras publicações” o que a literatura fala sobre esses planejamentos e respostas? O que foi diferente ou semelhante ao vivenciado pela equipe do relato?

- Discutir também as estratégias de avaliação de ações implementadas. Qual a relevância disso para a qualidade do sistema de saúde?

Recommendation: Minor revision

